# Transfer of Intracellular HIV Nef to Endothelium Causes Endothelial Dysfunction

**DOI:** 10.1371/journal.pone.0091063

**Published:** 2014-03-07

**Authors:** Ting Wang, Linden A. Green, Samir K. Gupta, Chul Kim, Liang Wang, Sharilyn Almodovar, Sonia C. Flores, Igor A. Prudovsky, Paul Jolicoeur, Ziyue Liu, Matthias Clauss

**Affiliations:** 1 Department of Microbiology & Immunology, Indiana University School of Medicine, Indianapolis, Indiana, United States of America; 2 Department of Cellular & Integrative Physiology and Indiana Center for Vascular Biology & Medicine, Indiana University School of Medicine, Indianapolis, Indiana, United States of America; 3 R. L. Roudebush VA Medical Center, Indianapolis, Indiana, United States; 4 Department of Medicine, Indiana University School of Medicine, Indianapolis, Indiana, United States of America; 5 Department of Medicine, Pulmonary Sciences & Critical Care Medicine, University of Colorado, Denver, Colorado, United States of America; 6 Center for Molecular Medicine, Maine Medical Center Research Institute, Scarborough, Maine, United States of America; 7 Institut de Recherches Cliniques de Montréal University of Montréal, Montréal, Quebec, Canada; 8 Department of Biostatistics, Indiana University School of Medicine, Indianapolis, Indiana, United States of America; Ghent University, Belgium

## Abstract

With effective antiretroviral therapy (ART), cardiovascular diseases (CVD) are emerging as a major cause of morbidity and death in the aging HIV-infected population. To address whether HIV-Nef, a viral protein produced in infected cells even when virus production is halted by ART, can lead to endothelial activation and dysfunction, we tested Nef protein transfer to and activity in endothelial cells. We demonstrated that Nef is essential for major endothelial cell activating effects of HIV-infected Jurkat cells when in direct contact with the endothelium. In addition, we found that Nef protein in endothelial cells is sufficient to cause apoptosis, ROS generation and release of monocyte attractant protein-1 (MCP-1). The Nef protein-dependent endothelial activating effects can be best explained by our observation that Nef protein rapidly transfers from either HIV-infected or Nef-transfected Jurkat cells to endothelial cells between these two cell types. These results are of *in vivo* relevance as we demonstrated that Nef protein induces GFP transfer from T cells to endothelium in CD4.Nef.GFP transgenic mice and Nef is present in chimeric SIV-infected macaques. Analyzing the signal transduction effects of Nef in endothelial cells, we found that Nef-induced apoptosis is mediated through ROS-dependent mechanisms, while MCP-1 production is NF-kB dependent. Together, these data indicate that inhibition of Nef-associated pathways may be promising new therapeutic targets for reducing the risk for cardiovascular disease in the HIV-infected population.

## Introduction

HIV-infected individuals have an increased risk of cardiovascular disease (CVD), even when successfully treated with virologically suppressive antiretroviral therapy (ART) [1]–[3]. This heightened risk persists even in the current treatment era, which uses antiretrovirals without the dysmetabolic effects (e.g. insulin resistance, dyslipidemia, hypertension) associated with earlier generation drugs. Importantly, HIV infection itself has recently emerged as an independent contributor to cardiovascular disease in this population [2], [4]–[6], which may be at least partially explained by the activities of the HIV virion envelope protein gp120 and the transcription activator Tat in endothelial cells [7], [8], and the synergistic interactions of these with HIV-induced cytokines, such as TNF-α [9]. Although anti-retroviral drugs were suspected to cause endothelial cell activation, a first study of suspected drug candidates did not reveal any of the anticipated endothelial activating effects [10]. This turned our attention to an intracellular HIV protein, Nef (Negative factor), which may be of particular relevance for those HIV-related cardiovascular patients on ART: Although HIV virion production is drastically reduced in these patients, HIV early gene expression, including Nef, is not affected to the same extent [11].

The HIV viral protein Nef is a small myristoylated protein devoid of enzymatic activity and its interactions with membranes and host cell proteins are central to its many effects, e.g responsible for T cell activation and enhanced virus production in vivo [12], which are believed to contribute to the broad HIV pathology and AIDS development [13]. The specific intracellular functions of Nef include alteration of protein trafficking and cell signaling cascades, inhibition of antibody maturation in B cells [14], and enhancement of HIV infectivity [15]. Nef has been shown to bind to Src homology-3 (SH3) domains of the Src family of kinases, thereby leading to signal transduction in T cells [16], [17] as well as alteration of membrane dynamics, resulting in an activated T cell state. Nef has been identified to induce the formation of both conduit-like nanotubes, which physically connect to bystander cells [18], and Nef-containing exosomes [19]. In infected monocytes, Nef induces nanotubes that can connect to B cells and mediate its own transfer to B cells where it inhibits Ig class switching [14], [20]. However, so far Nef transfer is only reported from infected to uninfected blood cells, and the possibility of Nef transfer to tissue cells has not been addressed.

Endothelial cells, especially in developing atherosclerotic plagues, are in direct contact with circulating HIV-infected cells and in a prime position for Nef transfer. Therefore, we hypothesize that Nef may also transfer to vascular endothelial cells and thus lead to endothelial activation, dysfunction and potentially progression to atherosclerosis. In this study, we propose a model in which Nef can mediate its transfer from Jurkat T cells to endothelial cells to trigger endothelial dysfunction. We further demonstrate that Nef contributes to endothelial dysfunction via two independent mechanisms, including (1) apoptosis of endothelial cells through an NADPH oxidase-dependent mechanism and (2) MCP-1 production through the NF-κB signaling pathway. Taken together, our study suggests inhibition of these Nef-induced pathways as a promising new therapeutic target for reducing the risk for cardiovascular disease in the HIV-infected population.

## Methods

### Reagents

HIV SF2 Nef, HIV NL4.3, HIV NL4.3-Nef deficient plasmids and Nef EH1 antibody were obtained from the NIH AIDS Reagent Repository. NADPH Nox2 inhibitor was a gift from Dr. Stephen Miller. Phalloidin-Cy5, live dye Vybrant DIO and JC-1 dye were obtained from Life Technologies (Grand Island, NY). Reverse transcriptase (RT) assay kit was purchased from Roche diagnostics (Indianapolis, IN). EDTA-free complete protease inhibitor mixture was obtained from Sigma (St Louis, MO) and 4–20% bis-Tris polyacrylamide gels were purchased from Thermoscientific (Walthem, MA). All other antibodies, Trolox, and apocynin were purchased from Abcam (Cambridge, MA). NF- κB inhibitor Ikki was purchased from R&D system (Minneapolis, MN). P65 siRNA was purchased from Cell Signal Technologies (Danvers, MA). All other reagents were obtained from R&D systems (Minneapolis, MN).

### Cell Culture

Human coronary arterial endothelial cells (HCAEC) were obtained from Lonza (Allendale, NJ) and maintained in culture medium consisting of basal medium EMB-2, 5% FBS, and supplemented with ingredients from Endothelial Cell Growth Media Kit (Lonza), including 0.4 ul/ml hydrocortisone, 4 ul/ml hFGF, 1 ul/ml VEGF, 1 ul/ml IGF-1, 1 ul/ml ascorbic acid, 1 ul/ml hEGF, 1 ul/ml GA-100 (gentamicin, amphotecerin B) and 1 ul/ml heparin. Jurkat cells and THP1 cells were purchased from American Tissue Culture Collection (ATCC, Manassas, VA) and cultured in RPMI 1640 medium supplemented with 10% fetal bovine serum and 100 U/ml penicillin. All primary cell cultures were maintained at 37°C in 5% CO_2_ and 95% air. Culture medium was changed every two days.

### Animals

Macaque heart tissues were obtained from a cohort of male Indian rhesus macaques (Macaca mulatta) infected with SHIVnefSF33, at 1000 TCID50 per animal [21]. The animals were housed at the California National Primate Research Center in accordance with the standards of the “Guide for the Care and Use of Laboratory Animals” and the American Association for Assessment and Accreditation of Laboratory Animal Care; necropsies were performed between 37–62 weeks post-infection, when the animals showed signs of immunodeficiency. Mouse heart tissues were obtained from University of Montreal of mice age 3 to 6 month kept according to institutional biosafety/AWA regulations and protocols. These transgenic mice have Nef and GFP or GFP alone expressed under the regulatory sequences of the human CD4 gene promoter. The experiments described in this study used formalin-fixed, paraffin-embedded heart tissues from these animals.

### HIV Replication Assay

HIV NL4.3 or HIV NL4.3-Nef deficient virus was produced by transfection of 293T cells. Transfection was performed with Lipofectamine LTX reagent (Invitrogen, Grand Island, NY), according to manufacturer’s instructions. Viral supernatant was harvested 48 h and centrifugated at 900 g for 10 min, to clarify the supernatant from remaining cells. Viral supernatant was used to infect Jurkat cells corresponding to a 10,000 cpm RT activity (equivalent to 1 ng/ml RT according to assay protocol (Roche diagnostics, Indianapolis, IN)). Virions in the supernatant were pelleted by centrifugation at 12,000 g for 1 hr and the RT activity was determined every other day for 9 days by the RT assay kit according to the manufacturer’s instructions (Roche, Indianapolis, IN). Infected cells were cocultured with HCAEC when virus titers were the same in both infected cells.

### Tissue Culture based Assays

For analysis using different pharmaceutical inhibitors, HCAEC were seeded in 6-well plates. Nef plasmid was transfected to HCAEC by Lipofectamine LTX reagent (Invitrogen, Grand Island, NY) (transfection efficiency ∼70% as determined by FACS). After 6 h post transfection, culture medium was changed, and inhibitors were added at optimized concentrations (200 nM Apocynin (Abcam), 200 nM Trolox (Abcam), and 100 nM Ikki (R&D Systems)). After a further 24 h, the supernatant was collected for MCP-1 production analysis as assessed by sandwich ELISA (Quantikine, by R&D Systems) and the cells were harvested for apoptosis analysis. For coculture working models, pcDNA only (mock control) or Nef-containing pcDNA plasmid was transfected into Jurkat cells by Lipofectamine LTX reagent. At 48 h post transfection, these Jurkat cells were cocultured with HCAEC in a 2∶1 ratio (In some cases, inhibitors were added at optimized concentrations as described). After 24 h coculture, the medium was centrifuged to remove Jurkat cells and the supernatant was collected for MCP-1 production analysis as assessed by sandwich ELISA (Quantikine, by R&D Systems). Any remaining Jurkat cells were washed off with PBS and further gated from endothelial cells by FACS based on forward scatter and side scatter profiles. HCAEC were harvested for apoptosis analysis.

### Detection of Apoptosis by Assessing DNA Fragmentation (TUNEL)

Apoptosis by terminal deoxynucleotidyl transferase-mediated dUTP nick end labeling in endothelial cells was performed as previously described [22]. Detection of apoptosis by FACS via a modified TUNEL approach was carried out using a fluorescence labeling system to detect dUTP end nicks according to the manufacturer’s instructions (APO-BRDU kit, Becton Dickinson).

### Confocal Immunofluorescence Microscopy

For in vitro studies, Nef and cDNA transfected Jurkat cells (transfection efficiency ∼20% as determined by FACS) were stained with cytoplasm live dye Vybrant DIO (Life Technologies, NY) (5 ul live dye/1 million cells at 37°C for 1 h), and cocultured with HCAEC for 24 h. Cells were fixed in 4% paraformaldehyde, permeabilized with 0.01% Triton X-100 in PBS (20 min at 4°C) and stained with 10 uM phalloidin and in some cases, anti-Nef EH1 antibody. Visualization was achieved by confocal microscopy (Olympus FV1000-MPE) with the same fluorescence intensities during image acquisition. The derived images were analyzed using MetaMorph software.

For staining mice and macaque heart sections, slides were de-paraffinized and processed as recently described [23]. Briefly, sections were stained with anti-GFP (for transgenic mice), or anti-Nef (EH1) (for macaque) antibody and endothelial-specific anti-von Willebrand factor (vWF) (1∶500 dilution) prior to staining with appropriate secondary fluorescent labeled antibodies (1∶1000 dilution). Visualization was achieved by confocal microscopy (Olympus FV1000-MPE) with the same fluorescence intensities during image acquisition.

### Detection of Reactive Oxygen Species (ROS) Activity by Dihydroethidium (DHE)

ROS production stimulated by Nef was determined using DHE as a fluorescent probe. Confluent HCAEC on 96-well black-walled dishes were incubated in conditioned BME medium and 5 µM DHE for 30 min to allow intracellular uptake. Cells were washed 3 times with PBS and media replaced with phenol-free RPMI. Fluorescence of the oxidized dye was subsequently determined at 520 nm (excitation), 605 nm (emission), with 590 nm cutoff on a Flex station set for maximal detection.

### NF-κB p65 Silencing in Endothelial Cells with Small Interfering RNA

For NF-κB p65 gene knockdown by RNAi we used Ambion’s Silencer® Select Custom Designed siRNA against p65 using a protocol as previously described [24]. Briefly, cells were transfected with Lipofectamine 2000 transfection reagent (Invitrogen) and after incubation for 2 days at 37°C, total cell lysate was used to determine the knockdown of p65 in human endothelial cells by Western blotting.

### Western Blot Analysis

Proteins were isolated from HCAEC using a cell lysis buffer consisting of 2.5 mM EDTA, 20 mM Tris pH 7.4, 100 mM NaCl, 1 mM Na_3_VO_4_, 1% Triton X-100, 10 mM NaF, 1% sodium deoxycholate, 0.1% SDS, 2.5 mM sodium pyrophosphate, 1 mM β-glycerol phosphate, and 1 tablet/10 ml EDTA-free complete protease inhibitor mixture (Sigma, St Louis, MO). Whole-cell extracts prepared from HCAEC were resolved in 4–20% bis-Tris polyacrylamide gels (Thermoscientific, Walthem, MA), followed by transfer to nitrocellulose membranes. Membranes were probed with anti-p65 antibody at 1∶2000 dilution in TBST buffer (Tris-Buffered Saline and Tween 20). Proteins were visualized by incubation with peroxidase-coupled secondary Abs in the presence of LumiGlo reagent while exposing in a Bio-Rad Chemidoc XRS/HQ.

### Monitoring Mitochondrial Function by JC-1 Dye-Mitchondrial Membrane Potential Probe

HCAEC were stained with 2 µg/ml JC-1 dye (Life Technologies) at 24 h post Nef transfection as recently described [25]. Briefly, JC-1 was dissolved in DMSO and Nef-transfected HCAECs were stained for 30 min at 37°C. The medium was replaced with PBS before the plate reading. Red fluorescence of the dye was determined at 455 nm (excitation), 590 nm (emission), with 590 nm cutoff on a Flex station (Molecular Device, Sunnyvale, California), set for maximal detection, whereas green fluorescence was determined at 485 nm (excitation), 538 nm (emission), with 530 nm cutoff. The ratio of red/green was determined as a measurement of mitochondrial depolarization.

### Data Acquisition and Statistical Analysis

MCP-1 production and apoptosis data were both expressed as fold increase normalized to the mean of control measurements (mock-transfected cells etc). Data are expressed as mean±SD for each group performed in triplicate and repeated at least three times. Data were analyzed with ANOVA and student’s t test. A p value <0.05 (marked as *) was considered statistically significant, and p<0.01 (marked as **) highly significant.

## Results

### Nef is Necessary for Endothelial Cell Activation and Cell Death

**Figure 1:**
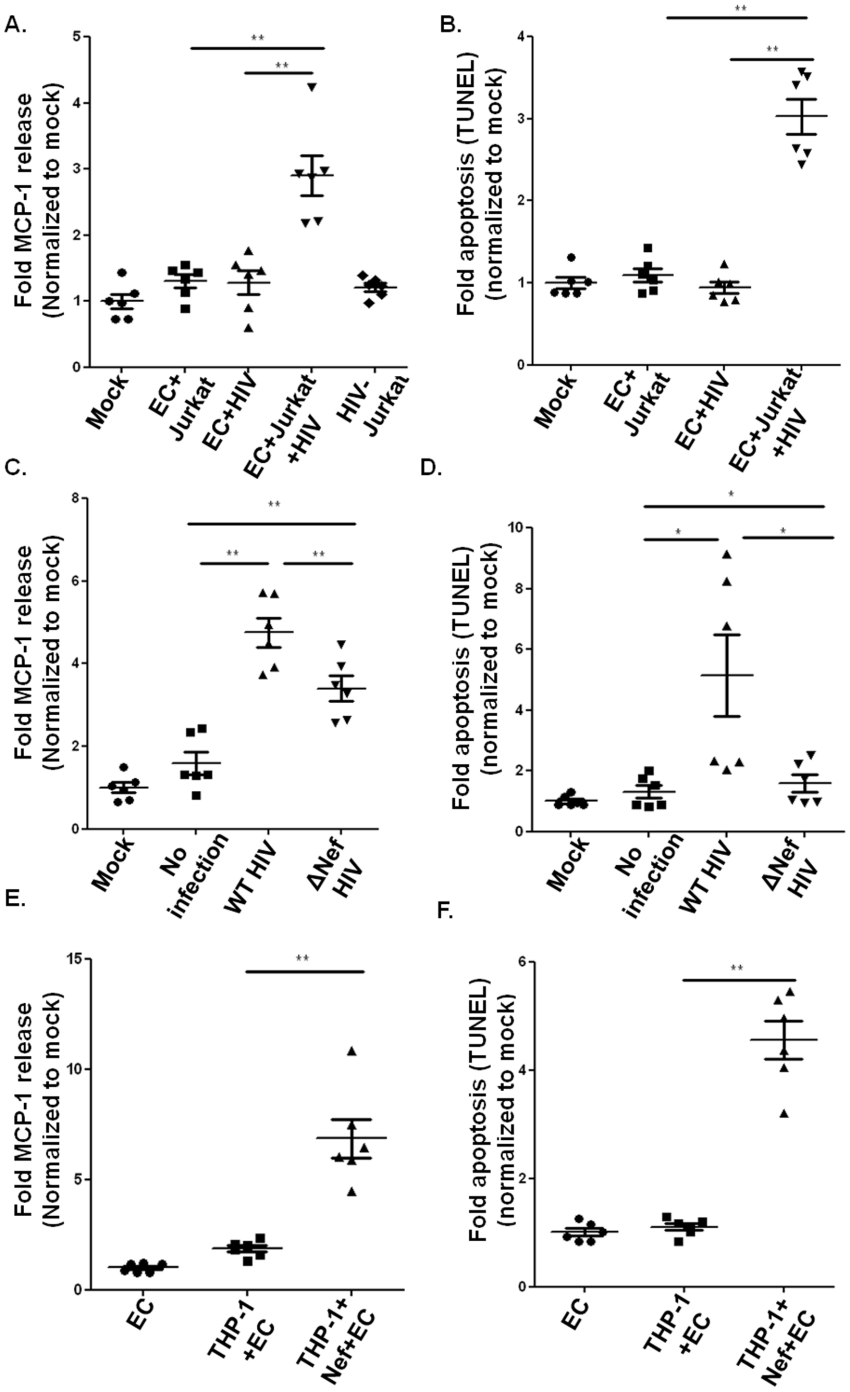
Nef is necessary for endothelial cell activation. A–B. 80% confluent endothelial cells were co-cultured in direct or indirect contact with HIV-infected Jurkat cells with HCAEC alone (Mock), HCAEC in direct contact with uninfected Jurkat cells (EC+T cell), EC in indirect contact with infected Jurkat cells (EC+HIV) or HCAEC in direct contact with infected Jurkat cells (EC+T cells+HIV). MCP-1 release from HIV infected Jurkat cells was also included (HIV-Jurkat). MCP-1 release was analyzed by ELISA (A); endothelial apoptosis was determined by TUNEL assay (B). C–D. MCP-1 release (C) and apoptosis (D) in endothelial cells were determined after co-culture of endothelial cells with uninfected Jurkat cells (mock), HIV-infected Jurkat cells (WT HIV) or Nef deleted HIV-infected Jurkat cells (ΔNef HIV). E–F. Endothelial MCP-1 production (E) and apoptosis (F) were determined in HCAEC alone (EC) or after 24 h coculture with cDNA (THP-1+ EC) or Nef transfected THP-1 cells(THP-1+ Nef+EC). Data were expressed as fold MCP-1 production and apoptosis, normalized to the mean of control measurements (N = 6. *P<0.05, and **P<0.01).

In viremic HIV patients, HIV envelope protein gp120 and transcription activator Tat are believed to mediate activation of vascular endothelium leading to endothelial dysfunction in pulmonary hypertension and cardiovascular diseases. However, intracellular proteins such as Nef have been not addressed in this context. To address the functional role of (intra)cellular versus extracellular proteins, we used a transwell filter system, which allowed us to compare direct cellular contact with indirect contact between HIV-infected Jurkat cells and endothelial cells. In this system, the bottom well containing HCAEC were either in direct contact with HIV- infected/uninfected Jurkat cells or separated by a semi-permeable membrane. As shown in Fig. 1A and 1B, HIV-infected Jurkat cells significantly increased endothelial MCP-1 production and apoptosis when in direct contact, while HIV-infected Jurkat cells separated from the endothelial monolayer exhibited minimal effect. We detected only a very minor MCP-1 release from HIV-infected Jurkat cells alone (Fig. 1A), indicating the observed increased MCP-1 production occurred from the endothelial cells and not Jurkat cells.

To specifically determine the involvement of Nef in these direct contact dependent effects, we employed wild type and Nef deleted HIV-infected Jurkat cells in co-cultures with HCAEC; while ensuring that virus titers were the same in both infected cells (Fig. S1). Jurkat cells infected with Nef deleted HIV induced a much weaker endothelial MCP-1 production and apoptosis in comparison to WT HIV (Fig. 1C and 1D), indicating that Nef protein is necessary for HIV-induced endothelial cell death and activation. These Nef dependent activities were also observed with Nef-transfected monocytes THP1 cells in close contact to HCAEC (Fig. 1E and 1F).

### Nef Enhances Live Dye Transfer from Jurkat Cells to Endothelial Cells via Direct Cell to Cell Contact

**Figure 2:**
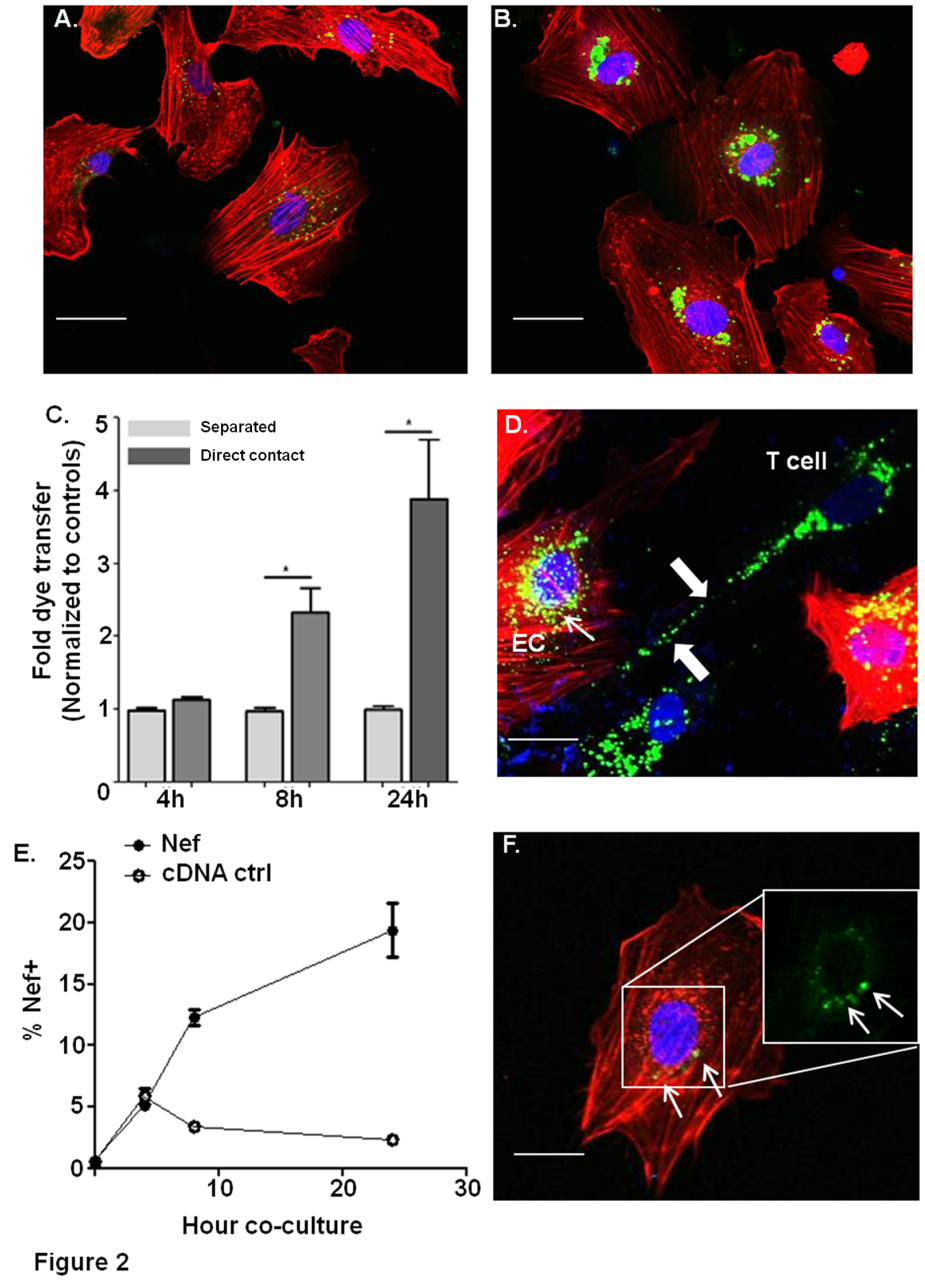
Nef enhances live dye transfer between cells and the formation of nanotubes. A–B. Live dye(green) transfer from cDNA transfected Jurkat cells (A) or Nef-transfected Jurkat cells (B) to endothelial cells after 24 h coculture. Endothelial cells (red) were stained by phalloidin. C. Live dye stained Nef or control cDNA transfected Jurkat cells were cocultured with HCAEC either in direct contact, or separated by transwell membranes for varying time points. Percentage of live dye transfer was determined and quantified by confocal microscopy. The fold dye transfer was normalized to cDNA controls for each condition. B is the representative figure form direct control experiments, which were quantified in C. D. Nanotube-like conduit formation (white arrows) between Nef transfected T cells labeled with live dye (green) and phalloidin labeled endothelial cells (red). E. Nef-transfected Jurkat cells were cocultured with HCAEC for varying time points, and HCAEC were stained for Nef to determine the time course of Nef transfer from Jurkat cells to endothelial cells. Endothelial cells were washed with PBS to ensure no adhesion of Jurkat cells. Any remaining Jurkat cells were gated from endothelial cells by FACS based on forward scatter and side scatter profiles. F. Nef transfer to HCEAC after 24 hr co-culture. Endothelial cells were stained with phalloidin (red) and Nef (green). Right corner insert indicates Nef accumulation in HCAEC without an overlay. Original magnification, X 60. Scale bars represent 10 µm.

Nef protein, but not virus, has been shown to be transferred to HIV-uninfected bystander cells [26]; therefore, we tested whether Nef protein could be transferred to endothelial cells. To achieve this, we labeled Nef or control cDNA transfected Jurkat cells with live dye (Vybrant DIO), then co-cultured with HCAEC and determined live dye transfer from Jurkat cells to HCAEC. At 24 hours coculture, Nef transfected Jurkat cells transferred significantly more dye into HCAEC (Fig. 2A, predominately in perinuclear regions) in comparison to cDNA transfected Jurkat cells (Fig. 2B). To determine how Nef induced live-dye transfer, we employed again the transwell filter system to distinguish between direct contact mediated and indirect contact transfer. We labeled Nef or control cDNA transfected Jurkat cells with live dye (Vybrant DIO), and observed direct but not indirect contact mediated live dye transfer relatively early after 8 hours (Fig. 2C). We also observed that Nef-transfected Jurkat cells formed multiple protrusions and nanotube like conduits towards HCAEC (Fig. 2D, arrow), which also displayed perinuclear live dye (Fig. 2D, arrowheads).

To confirm that this transfer of live dye to the endothelial cells includes Nef protein transfer, we co-cultivated HCAEC with Nef-transfected Jurkat cells and stained for intracellular Nef. Using FACS analysis, we found undetectable levels of transfer after 4 hours, but ca 9% and 17% of Nef positive HCAEC after 8 and 24 hour co-culture, respectively (Fig. 2E). These cells accumulated significant Nef staining at an ER-like/perinuclear location in endothelial cells after 24 h coculture with Nef transfected Jurkat cells (Fig. 2F, white arrowheads). Together, these data suggest that in our model Nef protein transfer from Jurkat cells to endothelial cells requires direct cell to cell contact.

### Nef Protein Presents in the Endothelium of an in vivo HIV Model

**Figure 3:**
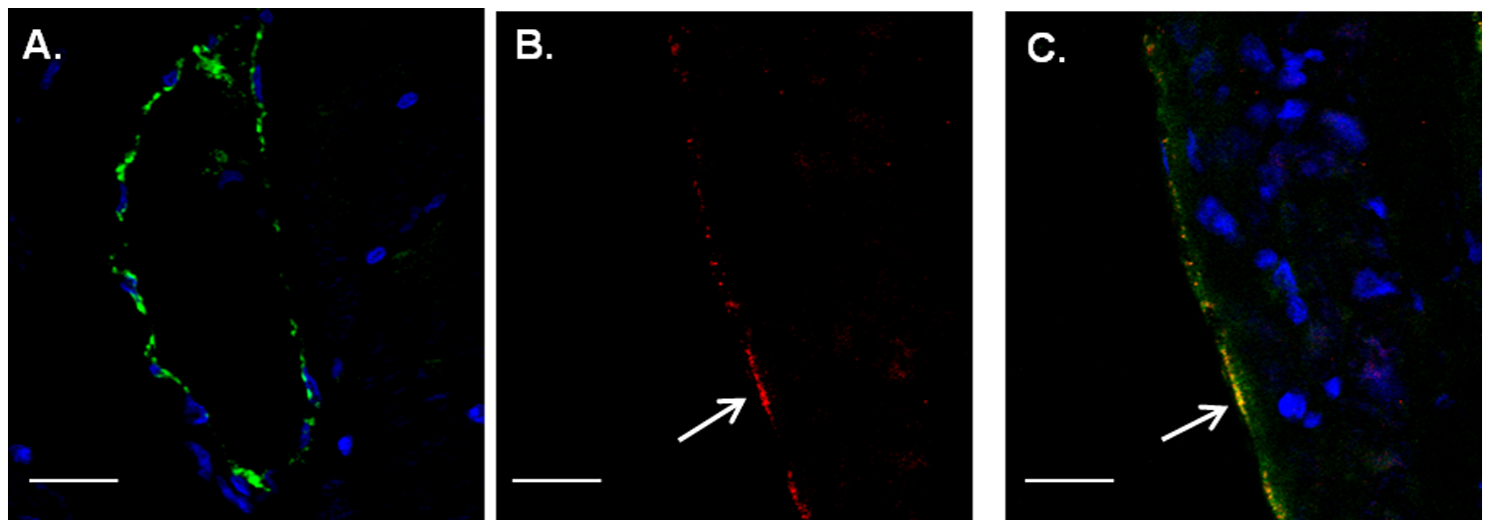
Nef is present in endothelial cells in vivo. A–C. Heart sections of single CD4-GFP (A) and double CD4-Nef-GFP (B, C) transgenic mice (N = 3; at least 4 pictures/slide) were double stained with GFP antibody (green, white arrows) and the endothelial marker vWF (red). Shown is GFP within the endothelial lining (arrows). Original magnification, X 60. Scale bars represent 100 µm. D–F. Macaque heart sections (N = 5; at least 4 pictures/slide) were double stained with IgG control (D) or Nef (E, F, red) and the endothelial marker vWF (green). Shown are cells double positive for Nef and vWF in coronary arteries (arrow). Original magnification, X 60. Scale bars represent 100 µm.

To access the potential relevance of Nef protein in coronary endothelial cells in vivo models, we first used transgenic mice in which Nef and GFP were expressed under the same regulatory sequences of the human CD4 gene, thereby resulting in Nef and GFP expression in CD4+ T cells and monocytes. Using double staining with anti-GFP and endothelial specific antibodies, GFP signal can be demonstrated in coronary vessels of CD4-Nef-GFP transgenic mice by confocal microscopy (arrows, Fig. 3B–3C) but not in single CD4-GFP Tg control mice supporting our results in vitro that Nef enhances the transfer of cytosol from T cells to endothelium (Fig. 3A). We then extended these results by using a chimeric simian immunodeficiency virus (SIV) expressing HIV Nef (SHIV Nef) macaque model. In this model, we detected Nef in coronary vessels (Fig. 3E–3F); vWF co-staining confirmed the presence of Nef in the endothelial lining (Fig. 3F, arrow, yellow overlay). Together these data provide in vivo evidence that Nef protein can target endothelial cells within the vascular system.

### Signal Transduction Analysis of Nef Induced Endothelial Activation and Dysfunction

**Figure 4:**
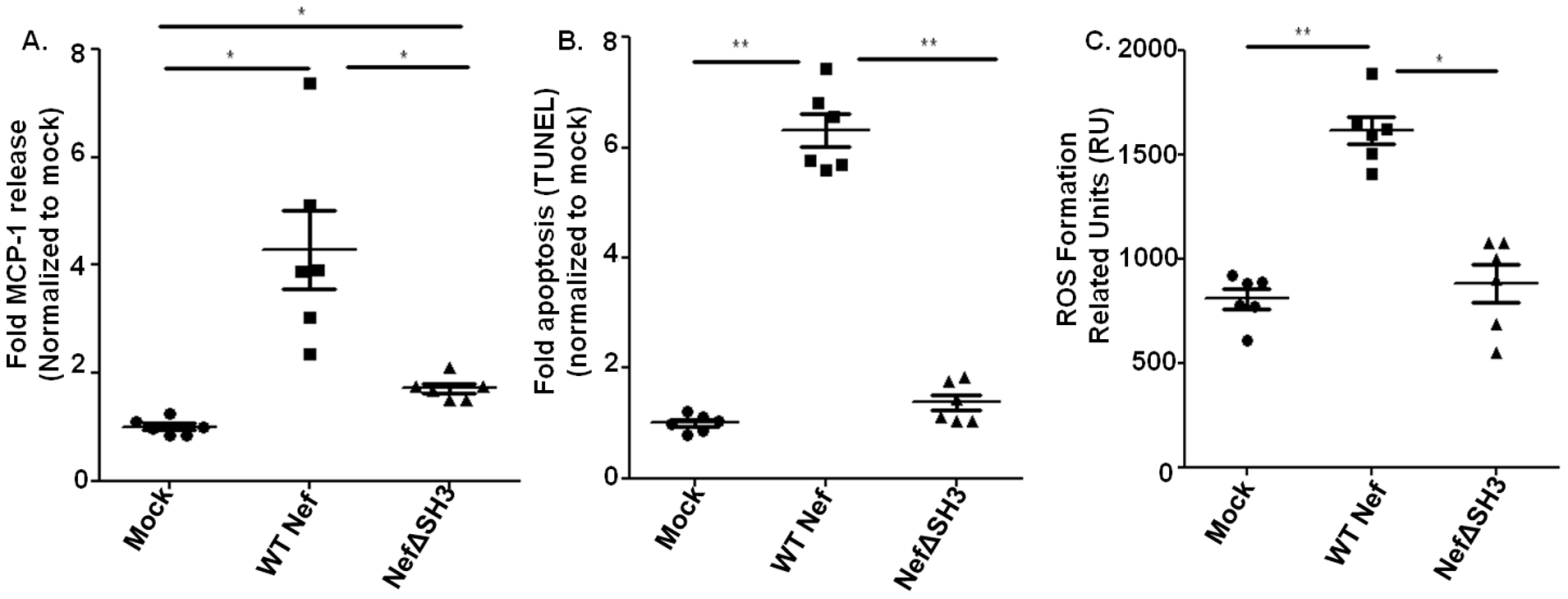
Nef is sufficient to induce endothelial activation and dysfunction. A–C. Endothelial MCP-1 release (A), apoptosis (B) and ROS formation (C) were determined in endothelial cells transfected with cDNA (mock), WT (WT Nef) or SH3 binding site mutated Nef (NefΔSH3) after 24 h. Data were expressed as fold MCP-1 production and apoptosis, normalized to the mean of control measurements (N = 6. *P<0.05, and **P<0.01).

**Figure 5:**
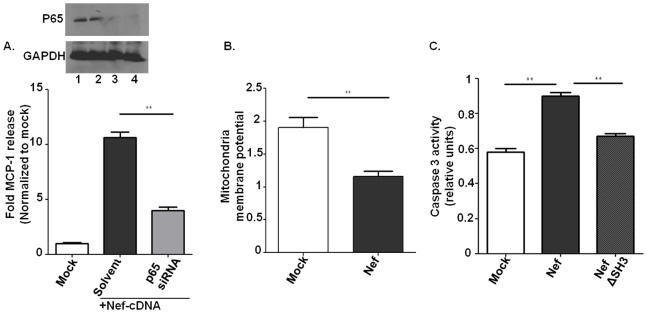
Nef induces endothelial activation and dysfunction through distinct pathways. A. 80% confluent endothelial cells were co-transfected with Nef cDNA and NF-κB p65 siRNA. After 18 h and 24 h, knock-down of p65 was confirmed by Western blot (Inserts: Lane 1, control; Lane 2, scrambled siRNA; Lane 3, 18 h p65siRNA knock-down; Lane 4, 24 h p65siRNA knock-down). MCP-1 production was tested at 24 h post Nef transfection in the supernatant using ELISA. B. Endothelial mitochondria potential was tested after 24 h post either control cDNA (mock) or Nef transfection by JC-1 staining kit according to the manufacturer’s instructions. C. Caspase 3 activity in endothelial cells was tested at 24 h post Nef transfection using a caspase 3 kit according to the manufacturer’s instructions.

To further analyze the signal transduction mechanism of intracellular Nef protein induced endothelial cell activation and dysfunction, we transfected Nef into HCAEC and measured endothelial MCP-1 release (Fig. 4A), apoptosis (Fig. 4B and Fig. 5C), mitochondrial dysfunction (Fig. 5B), and ROS formation (Fig. 4C), which were significantly increased after 24 h Nef transfection as compared to the cDNA controls. To address the Src homology-3 (SH3) domain of Nef, which is essential for many functions in T cells [27], [28], we also transfected HCAEC with SH3 domain mutated Nef. As shown in Fig. 4A–C, the SH3 binding site in Nef is essential for endothelial apoptosis induction and ROS formation, and somewhat important for MCP-1 production.

**Figure 6:**
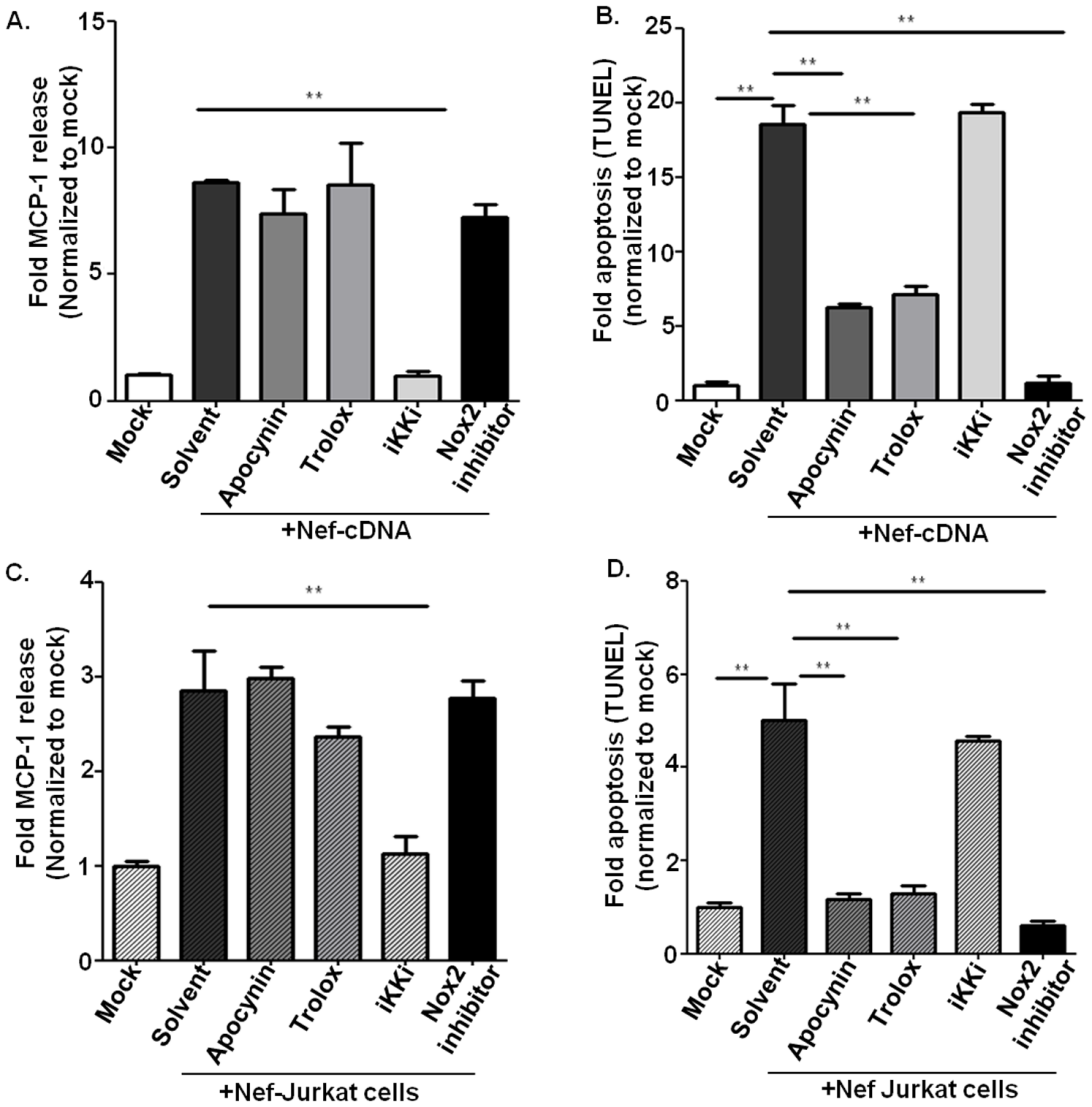
Potential pharmacological targets of Nef activities in endothelial cells. A–B. Endothelial cells were transfected with Nef cDNA, incubated for further 6 hours and treated with apocynin (200 nM), trolox (200 nM), Nox2 inhibitor (1 uM) or IKKi (100 nM). After additional 18 hours supernatants were analyzed for Nef-induced MCP-1 production (A) and endothelial cells for apoptosis using TUNEL (B). C–D. Endothelial cells were cocultured with Nef-transfected Jurkat cells for 24 h, and then treated with apocynin (200 nM), trolox (200 nM), Nox2 inhibitor (1 uM) or IKKi (100 nM) and incubated an additional 18 h, then analyzed for Nef-induced MCP-1 production (C) and apoptosis of endothelial cells (D). Data were expressed as fold MCP-1 production and apoptosis, normalized to the mean of control measurements. Data represent mean±SD from 3 separate experiments in which measurements were made in triplicate. *P<0.05, and **P<0.01.

In the following we aimed to specifically analyze the signal transduction pathway of Nef-induced HCAEC activation and dysfunction. First we tested involvement of NF-κB signaling for Nef-induced MCP-1 production. Using Ikki for canonical NF-κB signaling pathway [9] and NF-κB p65 siRNA we could demonstrated an essential role of NF-κB for Nef-induced chemokine production (Fig. 5A and 6A). However, NF-κB inhibition (Ikki) did not reduce Nef-induced endothelial apoptosis, indicating that Nef-induced apoptosis does not require NF-κB activation (Fig. 6B). Based on previous reports linking Nef to reactive oxygen species (ROS) formation [29], we addressed the role of ROS in Nef-induced endothelial dysfunction. Whereas the ROS inhibitor vitamin E derivative Trolox(Fig. S2) had no effect on Nef-induced MCP-1 production (Fig. 6A), Nef-induced apoptosis in HCAEC was completely abolished by Trolox (Fig. 6B). We further examined if Nef-induced ROS production and apoptosis is derived from NADPH oxidase using the antioxidant/NADPH oxidase inhibitor apocynin and the specific NADPH oxidase (Nox2) inhibitor, the gp91phox/Nox2 B-loop peptide (Nox2ds) [30]. Both inhibited Nef-induced ROS production (Fig. S3) and endothelial apoptosis (Fig. 6A). Furthermore, to rule out the possibility that Nef induced endothelial dysfunction is due to artificially high Nef concentrations caused by overexpression in endothelial cells, we used the Nef co-culture based transfer system as described above, in which Nef protein shuttles from Nef transfected Jurkat cells into cocultured HCAEC. We demonstrated the same signaling pathways for endothelial MCP-1 production and apoptosis in these HCAEC cocultured with Nef-transfected Jurkat cells (Fig. 6C and 6D).

## Discussion

The main finding of our study is that Nef protein induces its own transfer from Jurkat T cells and THP1 monocytes to endothelial cells, which consequently and necessarily induces endothelial cell activation, dysfunction, and death. Nef protein induces apoptosis of endothelial cells through an NADPH oxidase- and ROS-dependent mechanism, while Nef-induced MCP-1 production is NF-κB dependent. This involvement of ROS in Nef-induced endothelial apoptosis is in line with the widely accepted connection between endothelial dysfunction/oxidative stress and risk of cardiovascular events in patients with coronary artery disease in general [31] and in HIV individuals specifically [32]. Interestingly, Nef protein was already linked to pulmonary hypertension and endothelial dysfunction [33], [34]. Our finding of Nef protein-induced ROS production could explain Nef-induced decreased NO levels and ER dysfunctions in pulmonary arteries [34]. Furthermore, our observation that Nef protein can increase endothelial MCP-1 production concurs with the important role of this chemokine in atherosclerosis. This finding is particularly noteworthy in light of a link between early atherosclerosis and MCP-1 levels in HIV patients [35], [36], as arterial inflammation in HIV patients is a common denominator and associated with a circulating marker of monocyte and macrophage activation [37].

In our tissue culture models, quantification by FACS analysis revealed that 17% of HCAEC had Nef protein transferred to them from Jurkat cells/THP1 cells after 24 hours coculture. Our data suggests that the mechanism of Nef protein transfer between blood cells and vascular endothelial cells involves cell to cell contact. Reportedly, using IHC from human tissue many Nef protein expressing bystander cells can be found in lymphatic vessels, and under the low shear stress conditions in these vessels cellular transfer may be easily mediated by nanotube transfer from infected cells [14]. Under shear stress conditions, exosome-mediated transfer would be an alternative mechanism for transfer. Importantly, we here applied a membrane staining live dye (Vybrant Dio), which excludes the possibility of Nef transfer via gap junction. Nanotube transfer from blood cells to the endothelium is possible despite shear stress as T cells and monocytes are in constant close contact to vascular endothelium as part of immuno-surveillance. In addition, HIV infection itself may cause proinflammatory conditions leading to increased adhesion of infected and noninfected Nef carrying T cells and monocytes [38], and Nef specifically may increase the duration of T-cell-endothelium interactions [39], thus favoring nanotube formation and Nef transfer.

Using CD4-promoter-directed transgenic Nef expressing transgenic mice, we provided first evidence that Nef can be transferred from T cells into endothelial cells in vivo supporting our in vitro finding that Nef protein can be readily transferred from infected or transfected Jurkat cells to endothelial cells. Our demonstration of GFP being present in endothelial cells only when expressed together with Nef (Fig. 3) demonstrates clearly that Nef can also lead to cytosol transfer to endothelial cells in vivo. Although “leaky” CD4 regulatory elements cannot be totally excluded, our Nef staining occurs at a relatively high intensity, which is usually not seen in expression caused by leaky promoters. In addition, another line of evidence is provided by the demonstration of Nef protein in coronary endothelial cells using our established monkey model of HIV infection, in which macaques were infected with SHIV-Nef, a SIV construct containing HIV Nef alleles [33], [40]. In this model endothelial location of Nef has already been demonstrated in stenotic pulmonary arteries but not in healthy arteries of uninfected macaques, indicating a role of HIV Nef in pulmonary hypertension [33], [40]. However, further confirmation with human tissue samples would be required in future studies.

To explore potential targets that could interfere with Nef-induced endothelial dysfunction we addressed the mechanism of Nef action. First, we employed the fact that Nef protein contains a conserved motif with the minimal consensus (PxxP) site for SH3-mediated protein-protein interactions. This SH3 domain was shown previously to be involved in many Nef activities [16]. Further, our demonstration that the Nef SH3 binding site plays a key role in Nef-induced endothelial apoptosis is in line with previous reports that Nef activates and induces NADPH oxidase [41], [42], which may also explain the known impairment of eNOS activity and reduced NO bioavailabilty in HIV-related vascular dysfunction [43], [44]. Indeed, we observed that Nef induces apoptosis of endothelial cells through an NADPH oxidase- and ROS-dependent mechanism that is independent of NF-κB activation. Here, we included the ROS inhibitor trolox, a vitamin E derivative [45], and NADPH oxidase inhibitors, apocynin and NOX2ds. Recently, Heumüller et al. [46] suggested that instead of being an NADPH oxidase inhibitor, apocynin is an antioxidant in endothelial cells because apocynin dimers cannot form without myeloperoxidase (MPO), which is not expressed in endothelial cells. However, earlier studies have shown apocynin dimers in endothelial cells [47] and MPO may be transferred into endothelial cells by a cytokeratin 1 pathway [48]. Other peroxidases in endothelial cells in vivo may also substitute for MPO, such as a requirement for exogenous peroxidase and hydrogen peroxide in order for apocynin to function as an NADPH oxidase inhibitor [49]. To further support our hypothesis that Nef-induced endothelial ROS production involves NADPH oxidase activation, we also employed a more specific NADPH oxidase inhibitor, NOX2ds peptide, in this study. This peptide, which contains a HIV-Tat sequence that allows it to cross membrane barriers, specifically inhibits O(2)(•−) production by binding to the vascular isoform of NADPH oxidase-Nox2 oxidase, but does not inhibit ROS production by either Nox1- or Nox4-oxidase [30].

Of note, these NADPH oxidase- and ROS inhibitors were added early (6 hours) after transfection with Nef cDNA followed by determination of MCP-1 release, mitochondrial dysfunctions, ROS and apoptosis after 18 hours. Although we could demonstrate mitochondrial dysfunction together with Nef-induced endothelial cell apoptosis at later time points, this early inhibition of NADPH oxidase using either pharmacological or biochemical peptide based inhibition strongly suggests that NADPH activation precedes mitochondrial dysfunction and apoptosis in endothelial cells.

Given our finding that Nef-induced ROS production is necessary for Nef-induced endothelial cell death, anti-oxidant supplementation may be a tangible approach to reduce CVD in this population. In fact, in non-HIV infected patients, long-term administration of antioxidant vitamins C and E improved coronary and brachial artery endothelial function in patients with coronary artery disease, while multivitamin supplementation of HIV positive women during pregnancy reduced hypertension [50], [51]. However, the beneficial effects of antioxidants for CVD in the HIV negative population are controversial [52], [53] and further post data analyses or new studies within the HIV positive population would be interesting. In contrast to endothelial apoptosis, we found that NF-κB inhibition but not NADPH or ROS inhibition strongly reduced MCP-1 production. This is in contrast to TNF-alpha induced MCP-1 release, in which both ROS and NF-κB are required for transcriptional activation [54]. We have chosen to study MCP-1 production in endothelial cells as a readout for proinflammatory endothelial activation and dysfunction because it has been linked to cardiovascular diseases in a series of human and mouse studies [55], [56] as well as with HIV infection [36], [57].

Together, the main significance of this study is that Nef can transfer to endothelial cells and has dramatic effects, including the release of atherosclerotic chemokines, ROS formation, mitochondrial dysfunction and apoptosis. Pharmacologic interventional studies are now needed to determine the effects of Nef pathway inhibition in addition to ART to improve endothelial function and reduce the risk of cardiovascular disease in those infected with HIV.

## Supporting Information

Figure S1 **ΔNef HIV doesn’t inhibit HIV production in Jurkat cells.** There was a slight delay in ΔNef HIV production but both ΔNef HIV and WT HIV production reach the peak on day 8 or 9 post infection (dpi).
(TIF)

Figure S2 **Trolox inhibit Nef-induced ROS.** Trolox dose determination to block Nef-induced intracellular ROS activation in HCAEC as assessed with DHE using a Flexstation, which allow specific detection of intracellular ROS formation. Data represent mean±SD from 3 separate experiments in which measurements were made in triplicate. *P<0.05, and **P<0.01.
(TIF)

Figure S3 **Apocynin and Nox2ds dose determination to block Nef-induced intracellular ROS activation in HCAEC.** Apocynin with different dosage and optimized 1 uM Nox2 inhibitor were separately added to Nef cDNA transfected HCAEC for 18 h incubation. Detection of intracellular ROS formation was assessed with DHE using a Flex station. Data represent mean±SD from 3 separate experiments in which measurements were made in triplicate. *P<0.05, and **P<0.01.
(TIF)
