# Supplementary figures and images for: Transfer of Intracellular HIV Nef to Endothelium Causes Endothelial Dysfunction
Source: PLoS One. 2014 Mar 7;9(3):e91063. doi: 10.1371/journal.pone.0091063 (PMC3946685; doi:10.1371/journal.pone.0091063)

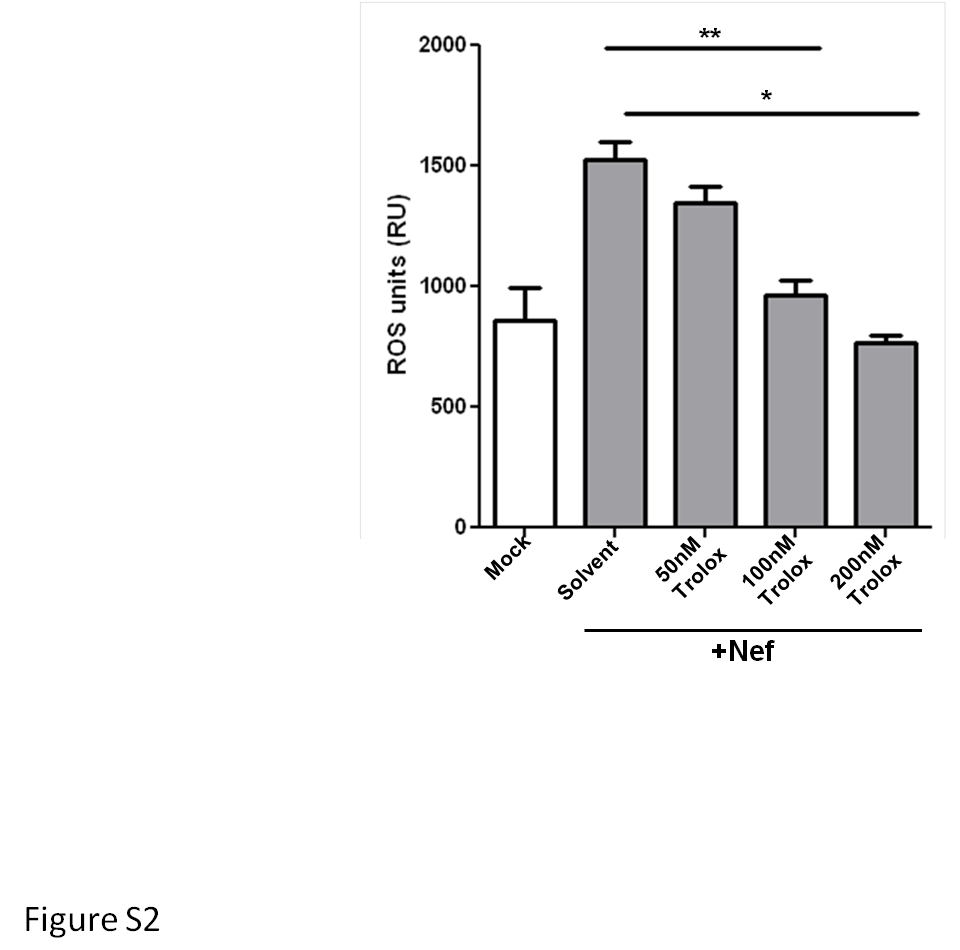

Supplement: Figure S2 — Trolox inhibit Nef-induced ROS. Trolox dose determination to block Nef-induced intracellular ROS activation in HCAEC as assessed with DHE using a Flexstation, which allow specific detection of intracellular ROS formation. Data represent mean±SD from 3 separate experiments in which measurements were made in triplicate. *P<0.05, and **P<0.01. (TIF) [file pone.0091063.s002.tif]
